# Differential Regulation of Oxidative Burst by First Line Drugs Used Against Multi Drug-Resistant Tuberculosis in Naïve Human Innate Immune Cells

**DOI:** 10.3390/antibiotics15060590

**Published:** 2026-06-09

**Authors:** Josephine Gal, Volda Gabro Stenback, Michaela Jonsson Nordvall, Thomas Schön, Robert Blomgran

**Affiliations:** 1 Division of Inflammation and Infection, Department of Biomedical and Clinical Sciences, Linköping University, 581 85 Linköping, Sweden; 2 Department of Clinical Microbiology, Region Östergötland, 581 85 Linköping, Sweden; 3 Department of Infectious Diseases, Linköping University Hospital and Kalmar County Hospital, Linköping University, 581 85 Linköping, Sweden

**Keywords:** tuberculosis, reactive oxygen species, non-specific effect, neutrophils, eosinophils, monocytes

## Abstract

**Background/Objectives**: Reactive oxygen species (ROS) are key effectors of innate immunity but can also contribute to inflammation and tissue injury when their production is dysregulated. Although antibiotics are primarily selected for their antimicrobial activity, prolonged treatment may also influence host immune responses. However, the effects of anti-tuberculosis drugs on ROS production across innate immune cell subsets have not been assessed, especially not for novel drugs currently used against multi drug-resistant (MDR) tuberculosis (TB). **Methods**: Whole blood from healthy donors was incubated with seven antimycobacterial drugs used against MDR TB at sub-therapeutic, therapeutic, and supra-therapeutic concentrations. ROS production was quantified by flow cytometry using dihydrorhodamine 123 (DHR-123) in neutrophils, classical monocytes, and eosinophils under unstimulated conditions or following stimulation with *Escherichia coli*, fMLP, or PMA. **Results**: Bedaquiline and clofazimine decreased ROS production in neutrophils and classical monocytes across multiple stimuli (median values of Rh-123^+^ classical monocytes after *E. coli* stimulation without BDQ was 30.8% versus 24.9% with 1 µg/mL BDQ (*p* < 0.05, therapeutic concentration) and 31.3% without CFZ versus 19.2% with 1 µg/mL CFZ (*p* < 0.01, therapeutic concentration) in the same conditions). In contrast, levofloxacin and linezolid showed no detectable impact on ROS production in any cell population. Pretomanid uniquely induced a reduction in ROS generation in eosinophils and classical monocytes while sparing neutrophil oxidative burst activity, distinguishing it from other antibiotics tested (34.1% decrease in Rh-123 MFI of eosinophils between the control and PA 3 µg/mL after fMLP stimulation, *p* < 0.01, therapeutic concentration). **Conclusions**: These findings demonstrate that first line drugs against MDR TB display heterogeneous and cell type-specific effects on innate immune oxidative responses. Such differential effects on host immunity may have implications for both antimicrobial efficacy and inflammation control during tuberculosis treatment.

## 1. Introduction

Innate immune cells can recognize, contain, and eliminate invading pathogens through phagocytosis, production of inflammatory mediators, and deployment of antimicrobial effector mechanisms such as reactive oxygen species (ROS). If both the pathogen and the host survive the initial interaction, the adaptive immune system is typically engaged to clear the infection. However, *Mycobacterium tuberculosis* (Mtb) is an example of pathogen capable of establishing persistent infection despite adaptive immunity. Mtb can persist within a privileged intracellular niche, the macrophage phagosome, allowing long-term survival within the host [1]. Persistent infection remains clinically asymptomatic, as in tuberculosis infection (TBI), yet carries a lifelong risk of reactivation into active disease.

Early after inhalation, Mtb bacilli encounter alveolar macrophages (AMs) [2]. Mtb is phagocytosed by AMs, which without an adaptive immune response are permissive to bacterial survival and replication, enabling establishment of infection. Infected AMs migrate from the airspace to the lung interstitium and produce pro-inflammatory cytokines and chemokines (e.g., IL-1β, IL-12, IL-6, IL-8, TNFα and IFN-γ) that recruit additional immune cells from the bloodstream, including neutrophils, monocytes, macrophages, and dendritic cells [2]. During the first days following infection, Mtb resides predominantly within AMs. By approximately 10–14 days post-infection in murine models, bacteria are distributed across multiple myeloid cell populations, including AMs, dendritic cells, and neutrophils, reflecting the recruitment and diversification of infected host cells [3]. As professional phagocytes, neutrophils and monocytes exert direct antimicrobial activity through phagocytosis, degranulation, cytokine and chemokine production, and ROS generation [4,5]. Neutrophils contribute to early bacterial control, in part through ROS-mediated antimicrobial activity. When activation becomes excessive or dysregulated, particularly during chronic or reactivating infection, this response is associated with increased neutrophil influx into lesions and can drive lung tissue damage and immunopathology [6]. Eosinophils, traditionally associated with allergic responses and helminth infections, have more recently emerged as potential contributors to TB immunity. Several animal models, including rhesus macaques, demonstrate early eosinophil migration to the lungs following Mtb infection [7,8]. Although their precise role remains incompletely understood, eosinophils can participate in antimicrobial defense, cytokine production, and immunoregulation, suggesting that they may influence early TB immune responses alongside neutrophils and monocytes.

ROS play a central role in innate immune responses to tuberculosis, acting as key antimicrobial effectors while also regulating immune signaling, metabolism, and cell death processes [9,10]. Although physiological ROS levels are essential for cellular function, excessive or sustained ROS production can exacerbate inflammation and drive tissue damage, highlighting the need for a tight regulation during infection. Tuberculosis treatment requires prolonged multidrug therapy [11], and while antibiotics are primarily selected for their bactericidal activity, growing evidence indicates that they may also modulate host immune functions [12,13]. Indeed, several antimicrobials have been shown to modulate host immunometabolic pathways. For example, antibiotics such as isoniazid and fluoroquinolones can influence inflammatory cytokine responses [14,15,16], while bedaquiline and other anti-tuberculosis drugs have been shown to activate host autophagy and innate immune pathways [17,18,19]. In addition, multiple agents, including bedaquiline, linezolid, and isoniazid, have been associated with alterations in mitochondrial function and cellular metabolism [20,21,22]. However, existing data remain fragmented, highly cell-type-dependent, and often limited to isolated cell populations.

In this study, we aimed to clarify the impact of anti-tuberculosis drugs on host innate immune oxidative responses. We assessed the effects of seven antimycobacterial agents mainly focusing on novel or repurposed drugs used against MDR TB, since data are particularly lacking regarding non-specific effects of these drugs. The antibiotics included represent distinct molecular targets and drug classes (Table 1). The panel includes agents with diverse mechanisms of action, such as ATP synthase inhibition (bedaquiline), protein synthesis inhibition (linezolid, amikacin), DNA synthesis inhibition (levofloxacin), and cell wall-targeting or redox-active compounds (clofazimine, pretomanid, and pyrazinamide). This diversity enables assessment of whether distinct antibacterial mechanisms differentially influence ROS production in innate immune cells.

Experiments were performed in whole blood to preserve physiological cell–cell interactions and to avoid artificial activation associated with isolation procedures. Intracellular ROS production was quantified by flow cytometry using a fluorescent oxidation-sensitive probe, dihydrorhodamine 123 (DHR-123) in eosinophils, neutrophils, and classical monocytes. Antibiotics were tested at sub-therapeutic, therapeutic, and supra-therapeutic concentrations, selected based on clinically relevant plasma peak levels and expected concentration gradients between two doses. To assess ROS generation through distinct activation pathways, cells were stimulated using receptor-mediated, phagocytic, and direct NADPH oxidase-activating stimuli. fMLP is a bacterial chemotactic peptide considered a relatively weak inducer of ROS, activating neutrophils through receptor-mediated signaling. *E. coli* stimulates ROS production mainly through phagocytosis, while PMA acts as a non-physiological, direct activator of NADPH oxidases and is widely used to elicit the full ROS-producing capacity of neutrophils. Using this approach, we aimed to characterize how anti-MDR tuberculosis drugs influence ROS generation in key innate immune cells under physiologically relevant conditions, thereby contributing to a broader understanding of how anti-tuberculosis therapies shape host oxidative responses, that may ultimately inform strategies to optimize bacterial clearance while limiting immune-mediated tissue damage.

## 2. Results and Discussion

### 2.1. Potent Suppression of ROS by Bedaquiline and Clofazimine Across Multiple Innate Immune Cell Types

**Figure 1:**
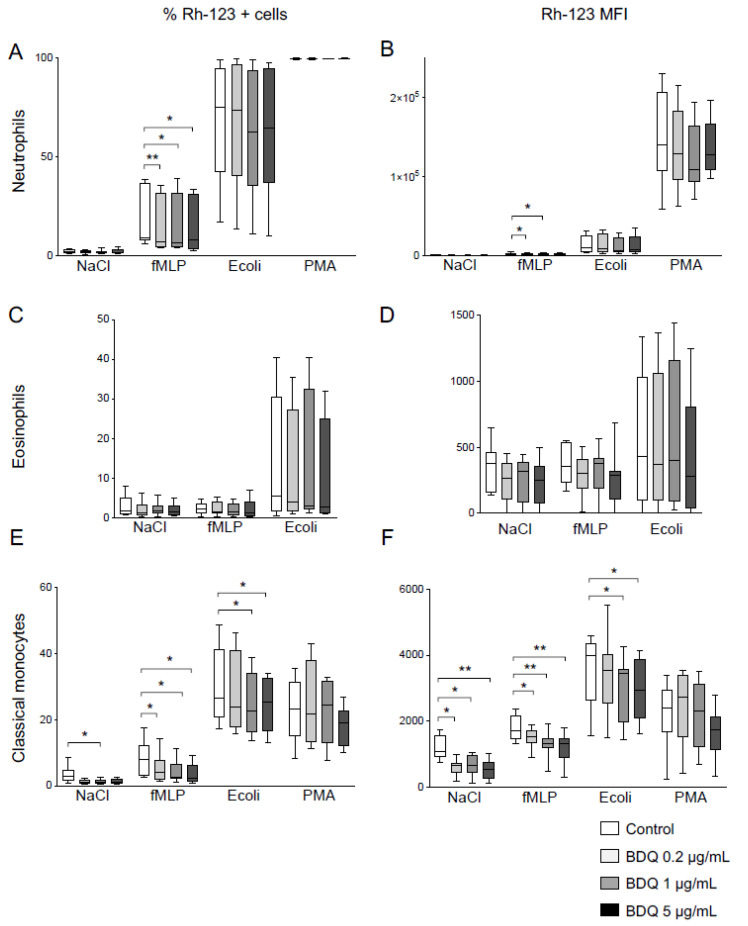
Effect of bedaquiline on ROS production in healthy donors (n = 9). (**A**) Percentage of Rh-123^+^ neutrophils. (**B**) Rh-123 MFI in neutrophils. (**C**) Percentage of Rh-123^+^ eosinophils. (**D**) Rh-123 MFI in eosinophils. (**E**) Percentage of Rh-123^+^ classical monocytes. (**F**) Rh-123 MFI in classical monocytes. Box shows 25–75 percentiles; whiskers show 10–90 percentiles. The line in the middle of the box is plotted at the median. Control represents the vehicle condition with a final DMSO concentration of 0.1%. Paired measures one-way ANOVA between the vehicle control and every antibiotic concentration for each stimulation separately, Tukey’s post-test. * *p* < 0.05 and ** *p* < 0.01.

**Figure 2:**
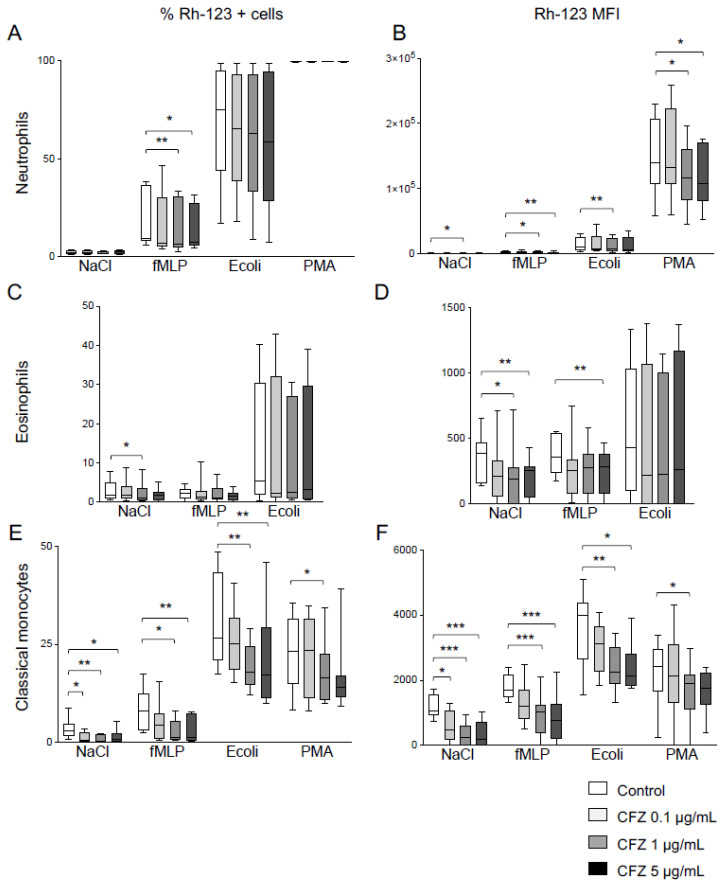
Effect of clofazimine on ROS production in healthy donors (n = 9). (**A**) Percentage of Rh-123^+^ neutrophils. (**B**) Rh-123 MFI in neutrophils. (**C)** Percentage of Rh-123^+^ eosinophils. (**D**) Rh-123 MFI in eosinophils. (**E**) Percentage of Rh-123^+^ classical monocytes. (**F**) Rh-123 MFI in classical monocytes. Box shows 25–75 percentiles; whiskers show 10–90 percentiles. The line in the middle of the box is plotted at the median. Control represents the vehicle condition with a final DMSO concentration of 0.1%. Paired measures one-way ANOVA between the vehicle control and every antibiotic concentration for each stimulation separately, Tukey’s post-test. * *p* < 0.05, ** *p* < 0.01, and *** *p* < 0.001.

Both bedaquiline (BDQ) and clofazimine (CFZ) are known to interfere with bacterial energy metabolism. They significantly reduced ROS production in neutrophils and classical monocytes across the different stimulation conditions tested. BDQ decreased both the proportion and mean fluorescence intensity of Rh-123^+^ neutrophils following fMLP stimulation (Figure 1A,B) (*p*-value = 0.025 between the vehicle control and BDQ 1 µg/mL for the percentage of Rh-123^+^ neutrophils), and similarly dampened ROS production in classical monocytes under unstimulated conditions as well as upon stimulation with fMLP or *E. coli* (Figure 1E,F). CFZ exerted even stronger suppressive effects, particularly in monocytes without or with all stimuli tested (Figure 2E,F). CFZ also markedly reduced ROS production in neutrophils (Figure 2A,B) and, unlike BDQ (Figure 1C,D), exerted a comparable downregulatory effect on eosinophils (Figure 2C,D).

These observations are consistent with the capacity of both drugs to modulate cellular metabolism and mitochondrial function. BDQ, originally characterized as an inhibitor of mycobacterial ATP synthase, has also been reported to alter host cell metabolism and mitochondrial activity. In human macrophages, BDQ has been shown to reprogram gene expression toward a bactericidal phenotype and enhance autophagy and lysosomal pathways, thereby enhancing intracellular bacterial clearance [19]. At the mitochondrial level in rat liver, BDQ can inhibit respiratory chain complex activities and decrease H_2_O_2_ production at high concentrations (above 27 µg/mL) [20]. Together, these findings support the notion that BDQ-mediated reduction in ROS observed here in neutrophils and monocytes may reflect interference with mitochondrial electron transport and oxidative metabolism. However, BDQ has also been reported to increase ROS in tumor cells [23], highlighting that ROS modulation by BDQ is context- and cell type-dependent.

CFZ has been associated with metabolic reprogramming toward glycolysis, reduced oxidative phosphorylation, and modulation of inflammatory cytokine responses in macrophages during *M. tuberculosis* infection [24]. However, its antimicrobial activity is commonly attributed to direct effects on the bacterium, where CFZ undergoes redox cycling and promotes the generation of ROS within *M. tuberculosis*, contributing to bacterial killing [25]. These distinct mechanisms highlight the difference between drug-host cell and drug-bacteria interactions, while the present study focuses on the former, CFZ-induced ROS production at the bacterial level may represent an additional, localized antimicrobial mechanism that is not captured in our experimental system. Our findings add to this growing evidence by showing that both drugs also modulate the oxidative burst capacity of key innate immune cells, potentially altering early host responses to infection and inflammation.

### 2.2. Pretomanid Reduced ROS Production in Eosinophils and Classical Monocytes

**Figure 3:**
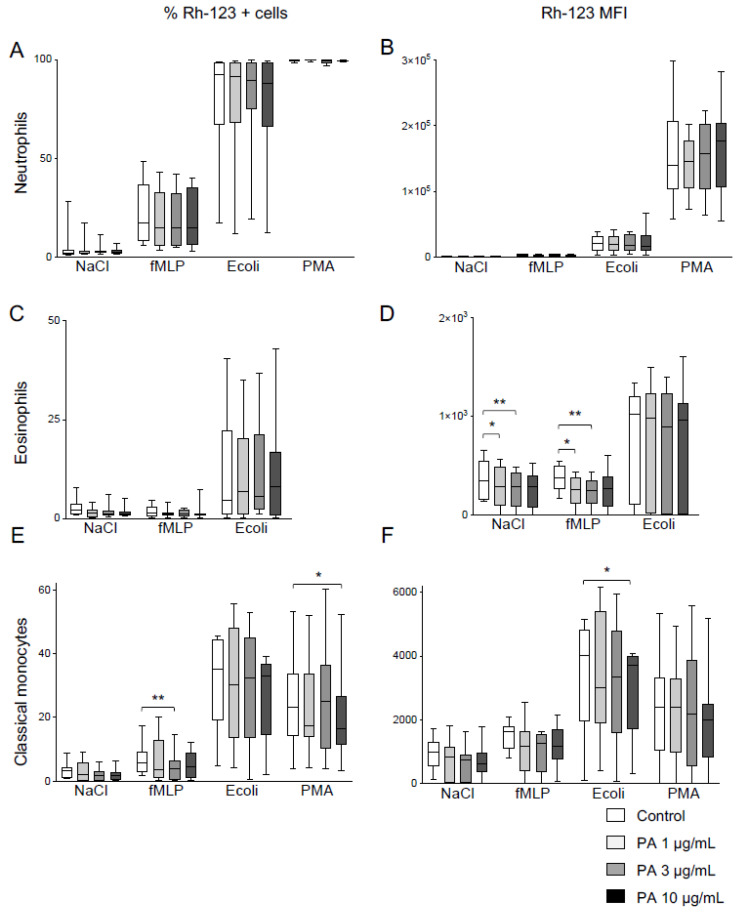
Effect of pretomanid on ROS production in healthy donors (n = 9). (**A**) Percentage of Rh-123^+^ neutrophils. (**B**) Rh-123 MFI in neutrophils. (**C**) Percentage of Rh-123^+^ eosinophils. (**D**) Rh-123 MFI in eosinophils. (**E**) Percentage of Rh-123^+^ classical monocytes. (**F**) Rh-123 MFI in classical monocytes. Box shows 25–75 percentiles; whiskers show 10–90 percentiles. The line in the middle of the box is plotted at the median. Control represents the vehicle condition with a final DMSO concentration of 0.1%. Paired measures one-way ANOVA between the vehicle control and every antibiotic concentration for each stimulation separately, Tukey’s post-test. * *p* < 0.05 and ** *p* < 0.01.

Pretomanid (PA), a cell-wall synthesis inhibitor, which also acts through inducing NO production when activated by *M. tuberculosis* [26], was associated with a modest decrease in ROS production in classical monocytes across all stimulation conditions tested (Figure 3E,F). A similar effect was observed in eosinophils, reflected by reduced Rh-123 mean fluorescence intensity, which reached significance both in the absence of stimulation (*p* = 0.009 between the vehicle control and PA 3 µg/mL) and following stimulation with fMLP (*p* = 0.006 between the vehicle control and PA 3 µg/mL) (Figure 3D). In contrast, no effect was observed in neutrophils (Figure 3A,B).

To our knowledge, data on the immunomodulatory actions of pretomanid are extremely limited, with most studies focusing on its bactericidal activity and synergy within novel TB regimens. The present results therefore suggest a novel role for PA in modulating host innate immune function through attenuation of ROS generation in monocytes but not neutrophils.

### 2.3. Levofloxacin and Linezolid Do Not Alter ROS Production in Innate Immune Cells

**Figure 4:**
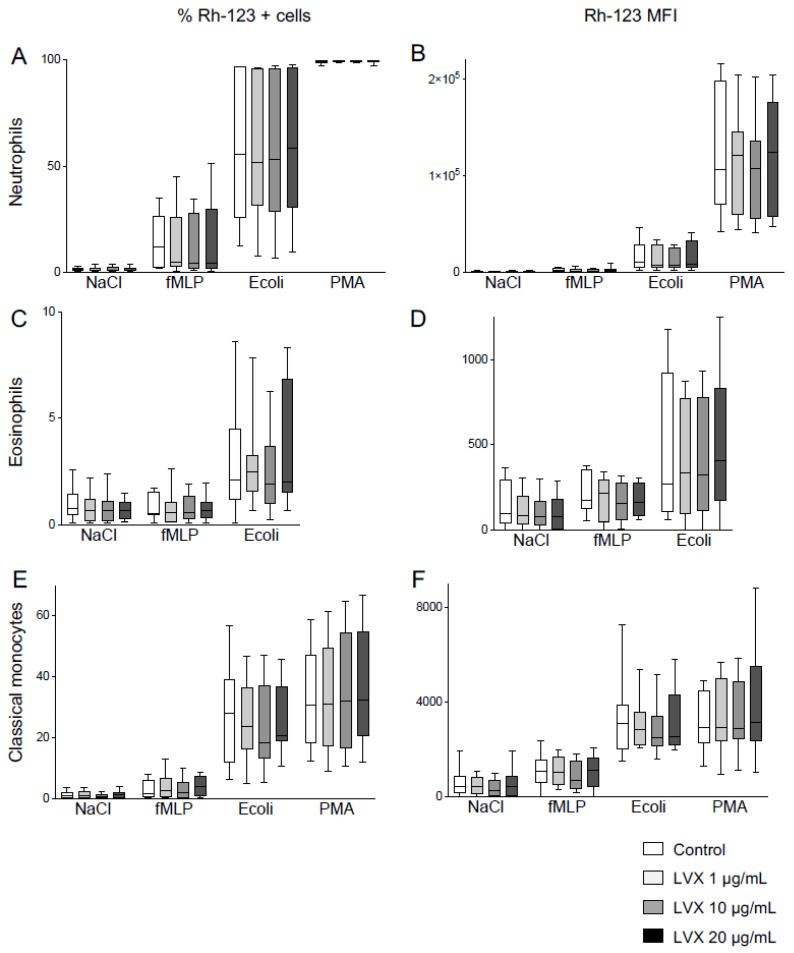
Effect of levofloxacin on ROS production in healthy donors (n = 9). (**A**) Percentage of Rh-123^+^ neutrophils. (**B**) Rh-123 MFI in neutrophils. (**C**) Percentage of Rh-123^+^ eosinophils. (**D**) Rh-123 MFI in eosinophils. (**E**) Percentage of Rh-123^+^ classical monocytes. (**F**) Rh-123 MFI in classical monocytes. Box shows 25–75 percentiles; whiskers show 10–90 percentiles. The line in the middle of the box is plotted at the median. Control represents the vehicle condition with a final H_2_O concentration of 0.1%. Paired measures one-way ANOVA between the vehicle control and every antibiotic concentration for each stimulation separately, Tukey’s post-test.

**Figure 5:**
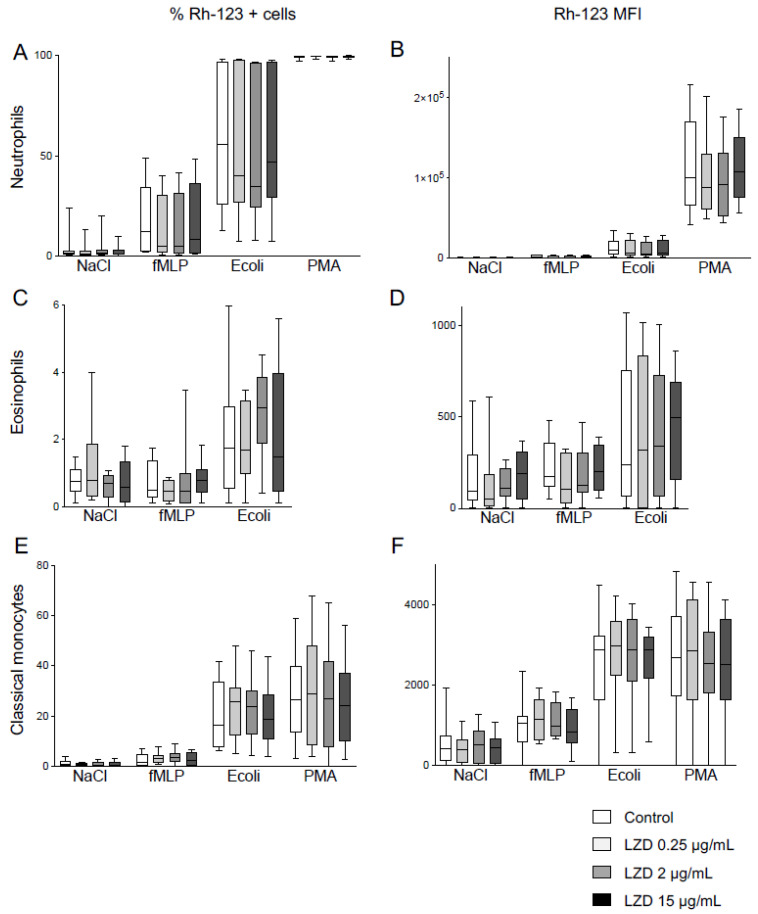
Effect of linezolid on ROS production in healthy donors (n = 9). (**A**) Percentage of Rh-123^+^ neutrophils. (**B**) Rh-123 MFI in neutrophils. (**C**) Percentage of Rh-123^+^ eosinophils. (**D**) Rh-123 MFI in eosinophils. (**E**) Percentage of Rh-123^+^ classical monocytes. (**F**) Rh-123 MFI in classical monocytes. Box shows 25–75 percentiles; whiskers show 10–90 percentiles. The line in the middle of the box is plotted at the median. Control represents the vehicle condition with a final H_2_O concentration of 0.1%. Paired measures one-way ANOVA between the vehicle control and every antibiotic concentration for each stimulation separately, Tukey’s post-test.

Both levofloxacin (LVX) and linezolid (LZD), which inhibit bacterial DNA replication and protein synthesis respectively, showed no detectable effects on ROS production in neutrophils, monocytes, or eosinophils under any of the conditions tested (Figure 4 and Figure 5). Neither the proportion of Rh-123^+^ cells nor the MFI changed meaningfully upon stimulation, indicating that oxidative burst capacity was preserved in the presence of these drugs.

This lack of effect on ROS is consistent with several previous reports, particularly for LZD. Multiple studies have shown that linezolid does not significantly alter phagocytosis, chemotaxis, or respiratory burst in polymorphonuclear neutrophils at clinically relevant concentrations [27,28]. Similarly, Grüger et al. (2012) reported no impact of LZD on killing or chemotactic behavior of neutrophils, reinforcing the idea that basic oxidative functions of innate immune cells remain intact [29]. Nevertheless, LZD is increasingly recognized as an immunomodulatory antibiotic despite leaving ROS responses here unchanged. It has been shown to modulate cytokine transcription and reduce production of TNF-α, IL-6, and IL-8 in several in vitro and in vivo models, with potential benefits in limiting excessive inflammation during infection [30,31,32,33]. Our findings therefore suggest that linezolid’s immunomodulatory actions occur independently from ROS generation.

For LVX, the literature reports more heterogeneous conclusions, with evidence of both ROS induction and inhibition depending on cell type, stimulus, and concentration. Fluoroquinolones have been shown to modulate cytokine production, and in some systems to promote mitochondrial ROS formation and apoptosis [34,35]. Other studies report enhanced hydrogen peroxide production or inhibition of superoxide under specific conditions [36,37]. Importantly, several investigations also demonstrate no significant change in oxidative burst in neutrophils [27,28], aligning with our present results. Taken together, these observations suggest that fluoroquinolone-mediated ROS modulation is highly context-dependent and did not manifest under the cellular conditions and stimuli used in our assays.

### 2.4. Limited Decrease in ROS Production in Neutrophils and Classical Monocytes by Pyrazinamide and Amikacin

**Figure 6:**
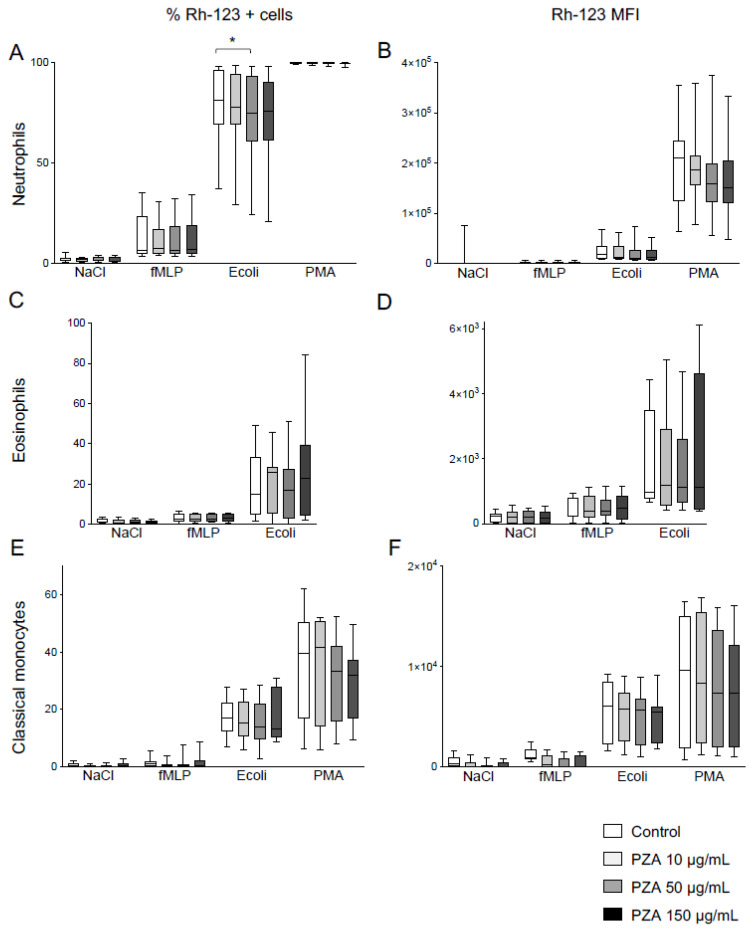
Effect of pyrazinamide on ROS production in healthy donors (n = 9). (**A**) Percentage of Rh-123^+^ neutrophils. (**B**) Rh-123 MFI in neutrophils. (**C**) Percentage of Rh-123^+^ eosinophils. (**D**) Rh-123 MFI in eosinophils. (**E**) Percentage of Rh-123^+^ classical monocytes. (**F**) Rh-123 MFI in classical monocytes. Box shows 25–75 percentiles; whiskers show 10–90 percentiles. The line in the middle of the box is plotted at the median. Control represents the vehicle condition with NaCl. Paired measures one-way ANOVA between the vehicle control and every antibiotic concentration for each stimulation separately, Tukey’s post-test. * *p* < 0.05.

**Figure 7:**
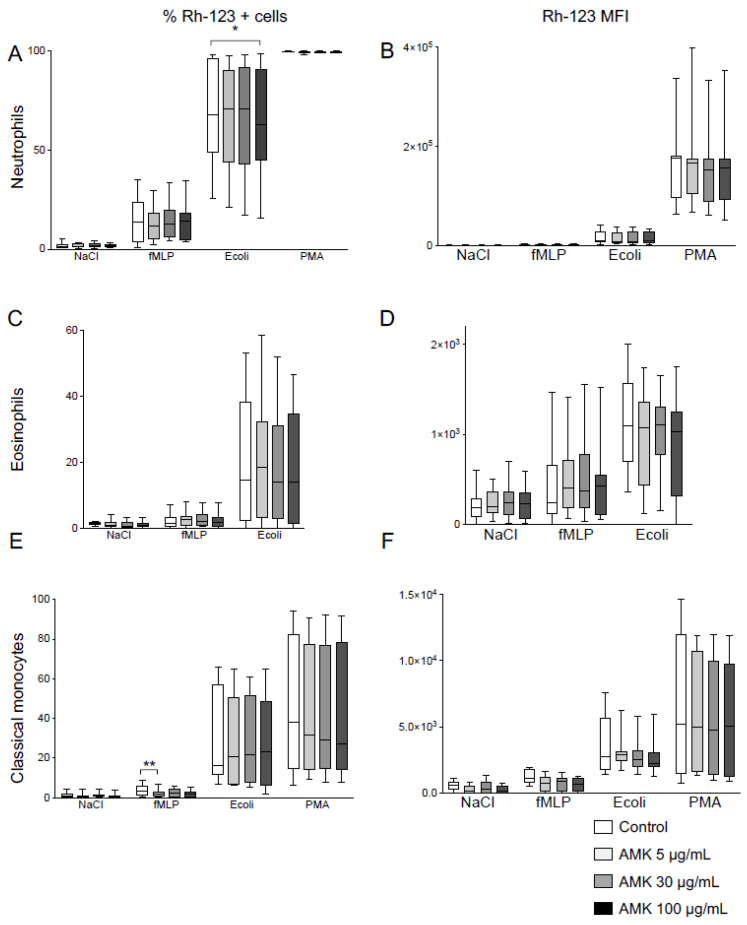
Effect of amikacin on ROS production in healthy donors (n = 9). (**A**) Percentage of Rh-123^+^ neutrophils. (**B**) Rh-123 MFI in neutrophils. (**C**) Percentage of Rh-123^+^ eosinophils. (**D**) Rh-123 MFI in eosinophils. (**E**) Percentage of Rh-123^+^ classical monocytes. (**F**) Rh-123 MFI in classical monocytes. Box shows 25–75 percentiles; whiskers show 10–90 percentiles. The line in the middle of the box is plotted at the median. Control represents the vehicle condition with NaCl. Paired measures one-way ANOVA between the vehicle control and every antibiotic concentration for each stimulation separately, Tukey’s post-test. * *p* < 0.05 and ** *p* < 0.01.

Pyrazinamide (PZA) and amikacin (AMK) exhibited comparable effects on ROS production in neutrophils and classical monocytes. Both drugs were associated with a reduction in ROS generation following stimulation, with significant changes detected in neutrophils when stimulated with *E. coli*. For PZA, neutrophils stimulated with *E. coli* demonstrated a significant decrease in the proportion of Rh-123-positive cells at 50 µg/mL compared to the condition without PZA (*p* = 0.026, Figure 6A). After PMA stimulation, treatment with 150 µg/mL pyrazinamide resulted in a 28.4% decrease in the median Rh-123 MFI compared to the PZA 0 condition (Figure 6B). Similarly, AMK resulted in significantly fewer Rh-123-positive neutrophils after *E. coli* stimulation at 100 µg/mL (*p* = 0.024, Figure 7A).

The observed reduction in ROS generation points to a potential immunomodulatory effect of PZA, in line with previous reports that antimicrobials can influence host immune cell functions. Interestingly, the direction of this effect contrasts with literature showing that PZA can modulate host inflammatory pathways by promoting autophagy and altering ROS production in Mtb-infected macrophages [17]. This contrast likely reflects differences in cell type (neutrophils and monocytes versus macrophages) and methodology (DHR-123 versus DCFH-DA and DHE assays). In addition, PZA has been reported to stimulate cytokine and nitric oxide production in murine macrophages, as well as in bone marrow-derived dendritic cells [38].

Consistent with the modest effects observed on oxidative burst in our assay, previous studies indicate that AMK does not induce broad activation of innate immune functions but instead exerts context-dependent and pathway-specific immunomodulatory effects. In human monocytes, AMK was shown to selectively induce the release of profibrotic mediators such as PDGF-AB and TGF-β1, while only modestly modulating TNF-α production depending on the activation state of the cells, and without affecting chemotaxis [39]. Our findings extend these observations by demonstrating that both drugs can significantly reduce ROS production in neutrophils under stimulation.

## 3. Materials and Methods

### 3.1. Ethics Statement

Verbal informed consent was obtained from all participants. Verbal consent was considered sufficient because the study exclusively involved anonymized surplus blood obtained during routine blood donation, without additional interventions or collection of identifiable personal information. Blood was obtained from healthy donors through the Linköping University Hospital Blood Bank. Anonymized samples were delivered to the researchers in accordance with the Declaration of Helsinki, not requiring a specific ethical approval according to paragraph 4 of Swedish law (2003:460) on Ethical Conduct in Human Research.

### 3.2. Antibiotics Preparation

Each antibiotic was prepared as a 100× stock solution and 5 µL was added to 500 µL of whole blood to achieve the indicated final concentrations (Table 1). A total of 10,000, 3000 and 500 µg/mL amikacin (A1774-1G, Sigma-Aldrich, Saint-Louis, MO, USA) and 15,000, 5000 and 1000 µg/mL pyrazinamide (P7136-100G, Sigma-Aldrich) were prepared in NaCl. A total of 1500, 200 and 25 µg/mL linezolid (PZ0014, Sigma-Aldrich) and 2000, 1000 and 100 µg/mL levofloxacin (077M4853V, Sigma-Aldrich) were prepared in dH_2_O. An amount of 500, 100 and 20 µg/mL bedaquiline (#HY-14881, MedChemExpress, Monmouth Junction, NJ, USA), 500, 100 and 10 µg/mL clofazimine (C8895-1G, Sigma-Aldrich) and 1000, 300 and 100 µg/mL pretomanid (SML-1290-10MG, Sigma-Aldrich) were prepared in DMSO. For each antibiotic, matched vehicle controls were included to account for any solvent-related effects and consisted of 0.1% DMSO, 0.1% dH_2_O, or NaCl, the latest being the main buffer of the assay. To assess potential interference of antibiotics with DHR-123 oxidation, a cell-free fluorescence assay was performed using horseradish peroxidase (HRP) and hydrogen peroxide (H_2_O_2_). In this control experiment, antibiotics or vehicle controls were added to wells containing HRP (0.5 U/mL final), H_2_O_2_ (100 µM final), and DHR-123 (5 µM final). Fluorescence was measured (Chameleon Microplate Reader, LabLogic, Chantilly, VA, USA) after a 4 min incubation at 37 °C to determine whether antibiotics affected DHR-123 activation. This approach enabled evaluation of whether the tested compounds directly induced and/or quenched DHR-123 fluorescence independently of the enzymatic oxidation system. While minor variations were observed in the cell-free system (Supplementary Figure S1), these were negligible and did not influence the conclusions drawn from the whole blood assay.

### 3.3. Intracellular ROS Production Assay

ROS production was assessed as previously described [40]. Briefly, sodium heparin (Na-heparin) whole blood was pre-incubated with different concentrations of TB antibiotics for 10 min at room temperature. A total of 100 µL of whole blood was transferred to FACS tubes and incubated on ice for 10 min before stimulation with 20 µL of either NaCl, fMLP (2.5 µM), PMA (4.05 µM), or *E. coli* K12 lyophilized cells reconstituted in NaCl (0.5 mg/mL). Dihydrorhodamine 123 (DHR-123), a membrane-permeable probe that is oxidized to fluorescent rhodamine 123 (Rh-123), was then added to detect intracellular ROS. Samples were incubated for 20 min in a water bath at 37 °C. To stop the reaction, tubes were placed on ice for 5 min. The antibody mix (CD125-PE from BD Biosciences, Fraklin Lakes, NJ, USA; CD193-PerCP/Cyanine5.5, HLA-DR-APC, CD14-BrilliantViolet 421, and CD16-BrilliantViolet 510 from BioLegend, San Diego, CA, USA; Fixable Viability Dye eFluor780 from ThermoFisher Scientific, Waltham, MA, USA; and Brilliant Stain Buffer from BD Biosciences) (Supplementary Table S1) was then added, followed by incubation for 10 min on ice and an additional 20 min at room temperature. As staining was performed directly in whole blood, no Fc receptor blocking step was included, as endogenous serum immunoglobulins reduce nonspecific Fc-mediated antibody binding [41]. Red blood cells were lysed, and after two washes, cells were resuspended in 200 µL of FACS buffer and acquired by flow cytometry. The percentage of Rh-123^+^ cells and the mean fluorescence intensity (MFI) of Rh-123 were determined for each cell population. Neutrophils were defined as SSChigh/CD16bright/HLA-DRlow/-, and eosinophils as SSChigh/CD16low/HLA-DRlow/-, CD193^+^, CD125^+^. Data from eosinophils stimulated with PMA were excluded from analysis due to marked phenotypic alterations. Monocyte subsets were identified as follows: classical monocytes (SSCintermediate/HLA-DR^+^/CD14^+^/CD16^−^), intermediate monocytes (SSCintermediate/HLA-DR^+^/CD14^+^/CD16^+^), and non-classical monocytes (SSCintermediate/HLA-DR^+^/CD14low/CD16^+^) (for the gating strategy see Supplementary Figure S1 in our previous work [42]). We herein focused our analysis on classical monocytes as they are present in reliable numbers for proper analysis of intracellular ROS production.

### 3.4. Flow Cytometry

Data acquisition was performed on a Gallios flow cytometer (Beckman Coulter, Brea, CA, USA) equipped with a 405 nm, 488 nm, and a 638 nm laser allowing detection of 10 colors. FlowJo v10 was used for data analysis.

### 3.5. Statistics

Statistical analyses were performed using GraphPad Prism version 10.5.0. Normality was assessed using the Shapiro–Wilk test. Depending on the distribution, significance testing was carried out using either a parametric test (one-way ANOVA) or a non-parametric test (Friedman test followed by Dunn’s post hoc test). Comparisons were made for each stimulation condition separately, testing each antibiotic concentration against the corresponding vehicle control. A *p*-value < 0.05 was considered statistically significant.

## 4. Conclusions

This study demonstrates that novel MDR TB drugs, such as bedaquiline and pretomanid, used as first line treatment in the BPaL [11] regimen (bedaquiline, pretomanid, linezolid) exert distinct immunomodulatory effects on oxidative burst responses in human innate immune cells. Using a whole-blood flow cytometry-based assay, we show that bedaquiline and clofazimine markedly suppressed ROS production across neutrophils and classical monocytes, with clofazimine exhibiting particularly strong inhibitory activity. Given the central role of ROS in microbial killing, immune signaling, and tissue damage, these results highlight the dual antimicrobial-immunomodulatory profile for these agents and may guide how to use them most efficiently during MDR TB. For example, CFZ may be useful to avoid early, ROS mediated tissue damage, at the same time as killing Mtb. Pretomanid exhibited a decrease in ROS production in classical monocytes, while notably sparing neutrophil oxidative responses. This distinctive profile suggests a potentially distinct immunomodulatory property of pretomanid and highlights cell type-specific regulation of oxidative pathways. To our knowledge, this represents the first indication that pretomanid may influence host redox biology, warranting further investigation.

Interestingly, neither levofloxacin nor linezolid altered ROS generation in neutrophils, monocytes, nor eosinophils under the conditions tested. This strengthens the results for BDQ and CFZ reducing ROS activity and the validity of the assay. Furthermore, the careful selection of clinically relevant drug levels in the experiment and the concentration-dependent effects on ROS production may increase the clinical relevance of the findings. Still, the clinical correlation of the level of changes in ROS production remains to be confirmed. It should be noted that for drugs like LZD and LVX, there is extensive data showing immunomodulatory effects on cytokine production, cell migration, and inflammatory signaling. These findings indicate that immunomodulation by these agents likely occurs through ROS-independent mechanisms and highlight the heterogeneity of host-directed effects among antimicrobial classes.

This study is limited by its focus on healthy donors, as prior results with isoniazid demonstrated divergent oxidative responses in latent tuberculosis-infected individuals compared to healthy controls [42]. In addition, donor demographic and clinical information was not available due to sample anonymization, preventing assessment of potential associations between inter-individual variability in ROS responses and biological variables such as age or sex. These differences highlight the need to extend immunomodulatory profiling of antimycobacterial drugs to patient populations including patients with active TB. Moreover, ROS production was assessed here exclusively using DHR-123, which reflects intracellular oxidant species and does not capture extracellular ROS release. Finally, the concentration dependence observed for several drugs emphasizes the need to determine whether similar modulation occurs at steady-state therapeutic levels or in relevant tissue compartments in vivo.

In conclusion, our findings on reduced ROS production from key MDR TB drugs support the hypothesis that antibiotics exert heterogeneous and drug-specific immunomodulatory effects on host innate immune cells. Differential regulation of oxidative burst responses may have important implications for how and when to use the drugs most efficiently, not only from the bacterial killing perspective but also regarding reducing immunopathology during tuberculosis therapy.

## Figures and Tables

**Table 1:** Overview of TB antibiotics used in the study, with corresponding plasma level, concentrations tested, class and molecular target.

Antibiotic	Plasma Level (C_max_)	Concentrations Tested	Class	Molecular Target
Pretomanid (PA)	2.3–4.3 μg/mL (43)	1 μg/mL	Nitroimidazoles	Aerobic conditions: inhibitor of a subfamily of mycolic acids (cell wall synthesis).
		3 μg/mL		Anaerobic conditions: inhibitor of the respiratory chain.
		10 μg/mL		
Clofazimine (CFZ)	0.5–2 μg/mL (43)	0.1 μg/mL	Riminophenazines	Cell wall: interferes with the proton-motive force and ATP production
		1 μg/mL		
		5 μg/mL		
Linezolid (LZD)	10–20 μg/mL (44)	0.25 μg/mL	Oxazolidinones	Protein synthesis inhibitor (anti-50S ribosomal subunit)
		2 μg/mL		
		15 μg/mL		
Levofloxacin (LVX)	8–13 μg/mL (45)	1 μg/mL	Fluoroquinolones	DNA synthesis inhibitor
		10 μg/mL		
		20 μg/mL		
Pyrazinamide (PZA)	20–90 μg/mL (46)	10 μg/mL	Nicotinamide synthetic derivative	Inner metabolism: translation inhibitor
		50 μg/mL		
		150 μg/mL		
Bedaquiline (BDQ)	1.4–2.9 μg/mL (47)	0.2 μg/mL	Diarylquinolines	Inner metabolism: ATP synthesis inhibitor
		1 μg/mL		
		5 μg/mL		
Amikacin (AMK)	15–35 μg/mL (48)	5 μg/mL	Aminoglycosides	Protein synthesis inhibitor (anti-30S ribosomal subunit)
		30 μg/mL		
		100 μg/mL		

## Data Availability

The original contributions presented in this study are included in the article/Supplementary Materials. Further inquiries can be directed to the corresponding author.

## Supplementary Materials

The following supporting information can be downloaded at https://www.mdpi.com/article/10.3390/antibiotics15060590/s1, Figure S1: Assessment of potential interference of antibiotics with DHR-123 oxidation. Effect of antibiotics on DHR-123 oxidation was assessed in a cell-free system containing horseradish peroxidase (HRP) and hydrogen peroxide (H_2_O_2_) in the presence of amikacin (AMK; NaCl vehicle), bedaquiline (BDQ; DMSO vehicle), clofazimine (CFZ; DMSO vehicle), levofloxacin (LVX; H_2_O vehicle), linezolid (LZD; H_2_O vehicle), pyrazinamide (PZA; NaCl vehicle), or pretomanid (PA; DMSO vehicle). Antibiotics were added at the start of the reaction, and endpoint fluorescence measurements were performed after 4 min, in a cell-free system otherwise reaching Rh-123 fluorescence maximum (plateau) after 8 min, facilitating detection of both increased and decreased signals at the 4 min time-point. Values are presented as mean ± SEM of three independent experiments. One-way ANOVA between every antibiotic and the corresponding vehicle control, Tukey’s post-test. Table S1: Antibodies’ details.

## Abbreviations

The following abbreviations are used in this manuscript:

AMK	Amikacin
BDQ	Bedaquiline
CFZ	Clofazimine
DHR-123	Dihydrorhodamine 123
fMLP	N-Formylmethionine-leucyl-phenylalanine
LVX	Levofloxacin
LZD	Linezolid
MDR	Multi drug-resistant
MFI	Mean Fluorescence Intensity
Mtb	*Mycobacterium tuberculosis*
PA	Pretomanid
PMA	Phorbol 12-myristate 13-acetate
PZA	Pyrazinamide
Rh-123	Rhodamine 123
ROS	Reactive Oxygen Species
TB	Tuberculosis
TBI	Tuberculosis infection
