# Supplementary material for: Differential Regulation of Oxidative Burst by First Line Drugs Used Against Multi Drug-Resistant Tuberculosis in Naïve Human Innate Immune Cells
Source: Antibiotics (Basel). 2026 Jun 9;15(6):590. doi: 10.3390/antibiotics15060590 (PMC13296184; doi:10.3390/antibiotics15060590)
Supplement: Supplementary file 1 [file antibiotics-15-00590-s001.zip › antibiotics-4343864-supplementary.pdf]

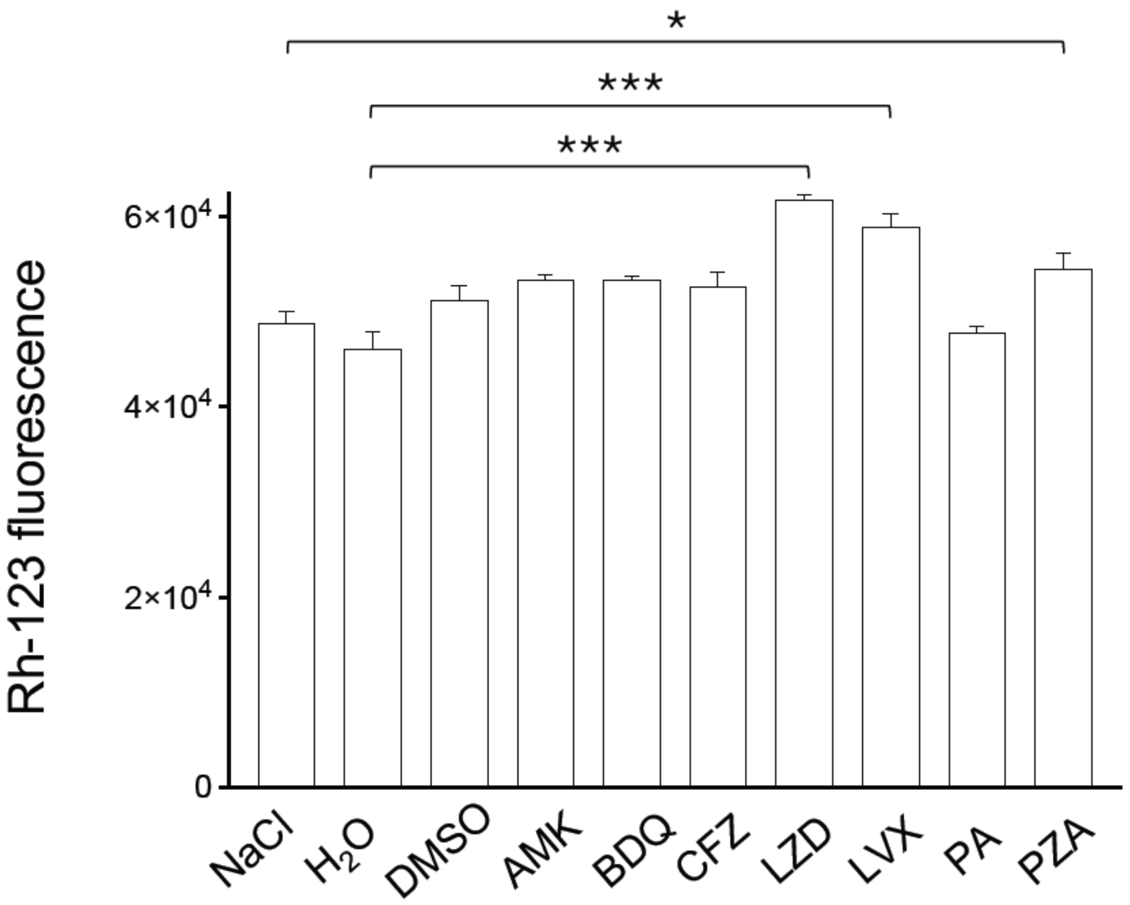

Figure S1: Assessment of potential interference of antibiotics with DHR-123 oxidation. Effect of antibiotics on DHR-123 oxidation was assessed in a cell-free system containing horseradish peroxidase (HRP) and hydrogen peroxide (H_2O_2) in the presence of amikacin (AMK; NaCl vehicle), bedaquiline (BDQ; DMSO vehicle), clofazimine (CFZ; DMSO vehicle), levofloxacin (LVX; H_2O vehicle), linezolid (LZD; H_2O vehicle), pyrazinamide (PZA; NaCl vehicle), or pretomanid (PA; DMSO vehicle). Antibiotics were added at the start of the reaction, and endpoint fluorescence measurements were performed after 4 min, in a cell-free system otherwise reaching Rh-123 fluorescence maximum (plateau) after 8 min, facilitating detection of both increased and decreased signals at the 4 min time-point. Values are presented as mean ± SEM of three independent experiments. One-way ANOVA between every antibiotic and the corresponding vehicle control, Tukey's post-test. * $p < 0.05$; *** $p < 0.001$

Table S1: Antibodies' details.

Target antigen	Clone	Vendor or Source	Catalog #
**HLA-DR**	L243	Biolegend	307610
**CD14**	HCD14	Biolegend	325628
**CD16**	3G8	Biolegend	302048
**CD193**	5E8	Biolegend	310718
**CD125**	A14	BD Biosciences	555902
